# The effect of therapeutic horseback riding on heart rate variability of children with disabilities

**DOI:** 10.4102/ajod.v5i1.248

**Published:** 2016-08-18

**Authors:** Zingisa Nqwena, Rowena Naidoo

**Affiliations:** 1 College of Health Sciences, University of KwaZulu-Natal, Durban, South Africa

## Abstract

**Background:**

Heart rate variability (HRV) is the oscillation in the interval between consecutive heart beats, resulting from dynamic interplay between multiple physiologic mechanisms that regulate instantaneous heart rate. Short-term heart rate regulation is governed by sympathetic and parasympathetic neural activity and therefore HRV examination can be used as a non-invasive estimate of the functioning of the autonomic nervous system (ANS).

**Aim:**

To determine the effects of therapeutic horseback riding (THR) intervention on the HRV of children with disabilities. The objective was to examine if THR intervention improves the HRV of children, hence improving the parasympathetic activity that is associated with a calm and relaxed state.

**Methods:**

This is a quasi-experimental design. Heart rate variability components were measured over six intervention sessions of THR. Heart rate variability measures were recorded from 29 participants with various disabilities, and was assessed in both time and frequency domains.

**Results:**

Over the six THR sessions, the time domain showed an increase in HRV for pre-THR indicating improved vagal activation, whereas frequency domain showed both increased sympathetic activity and increased parasympathetic activation during THR based on different components of frequency domain.

**Conclusion:**

Therapeutic horseback riding intervention of six sessions demonstrated a change in HRV of children with disabilities. However, the changes obtained were not significant to make conclusive measures as to whether sympathetic or parasympathetic activity is predominantly increased after the six sessions. Further research involving more than six sessions of THR is required to yield more significant changes.

## Background

Therapeutic horseback riding (THR) is a method of treatment involving riding a horse and performing certain activities on a horse to accomplish physical, emotional, social, cognitive, behavioural and educational goals (Lessick *et al*. 2004). THR is similar to hippotherapy. However, THR is performed by a trained instructor teaching the rider to improve basic riding skills whilst hippotherapy is performed by a physical therapist or occupational therapist using equine movements to improve posture, balance and fine motor skills (Snider *et al*. 2007).

THR is one of the methods used as a treatment and management tool for children with disabilities, including cerebral palsy, learning disabilities, amputations, autism, spinal cord injuries, neurological disorders and emotional problems (Lessick *et al*. 2004). The movement of the horse’s pelvis moves the rider with the same three dimensional movements which occurs during human walking, providing motor and sensory inputs similar to those received during walking, hence providing neuromuscular stimulation (Bowes & Cook 2007). The benefits of THR include stimulation of respiration and circulation, improved range of motion, and relaxation of tight muscle for conditions such as muscular dystrophy, poliomyelitis, amputation and multiple sclerosis (Riding for the Disabled Association 1987). Studies have also shown improvements in balance (Homnick *et al*. 2015; Kang 2015; Miller & Alstan 2004), motor and sensory efficiency (Wuang *et al*. 2010) and social motivation (Bass, Duchowny & Llabre 2009).

Numerous benefits of THR have been proven through various methods (Homnick *et al*. 2015; Kang 2015; Miller & Alstan 2004); however, the use of heart rate variability (HRV) as a method to measure stress or relaxation status is limited. HRV can be used to assess if an individual is stressed or is in a more relaxed state (Michels *et al*. 2013).

HRV is the oscillation in the interval between consecutive heart beats (Schroeder *et al*. 2004). Heart rate (HR) varies from beat to beat during normal sinus rhythm (Bilchick & Berger 2006). HRV can simply be defined as a time gap between the heart beats, which normally varies throughout the respiratory cycle. It is measured by calculating the time between the R peaks on an electrocardiogram (ECG) trace (Taskforce 1996). Low HRV is defined as a low variability in the distance between consecutive heart beats, whilst HRV is a high variability in the distance between consecutive R peaks of the heart beat signal (Michels *et al*. 2013). In healthy individuals, HRV is normally higher, whilst lower HRV is associated with increased risks of cardiovascular diseases (Colhoun *et al*. 2001).

HRV results from the dynamic interplay between multiple physiologic mechanisms that regulate instantaneous HR (Bilchick & Berger 2006). Short-term HR regulation is governed by sympathetic (fight or flight) and parasympathetic (relaxation state) neural activity (Bilchick & Berger 2006) and, therefore, HRV examination can be used as a non-invasive estimate of the functioning of the autonomic nervous system (ANS). Low parasympathetic activity is linked to poor emotion regulation (Porges, Doussard-Roosevelt & Maiti 1994) and high stress levels (Porges 1995). Furthermore, HRV can also be used to determine regulation of the peripheral viscera and the heart by the ANS (Xie *et al*. 2005).

HRV has time and frequency domain components. Time domain includes the mean inter-beat interval (R-R interval), which is the distance between successive heartbeats, expressing mainly the parasympathetic activity. The mean squared successive differences (MSSD) between R-R intervals is the estimate of the short-term component of HRV and provides vagal index. An increase in this value also reflects an increase in parasympathetic activity (Taskforce 1996). This value can also be calculated as a root mean squared successive difference (RMSSD) between R-R intervals. Total power (TP) is the variance of all the R-R intervals, with frequency ranges approximately ≤ 0.4 Hz, measured in ms^2^ (Taskforce 1996). Component coefficient of variation (CCV) also represents the variation of HRV (expressed in %). CCV high frequency (HF) and CCV low frequency (LF) reflect the parasympathetic and sympathetic activity, respectively (Kurosawa *et al*. 2007).

Frequency domain measures pertain to HRV at certain frequency ranges associated with specific physiological processes. Parameters evaluated are TP at HF and LF. The TP at HF peak (0.15–0.4 Hz) corresponds to respiratory sinus arrhythmia and reflects the parasympathetic activity (Moodithaya & Avadhany 2009) and the TP at LF peak (0.04–0.15 Hz) predominantly reflects sympathetic activity as it is influenced by blood pressure baroreceptor mediated regulation (Moodithaya & Avadhany 2009). The LF/HF ratio is also used to assess the balance between sympathetic and parasympathetic activity. An increase in the ratio indicates an increase in sympathetic activity and a decrease indicates a predominant increase in parasympathetic activity.

The variables of interest in this study were power at HF, power at LF, LF/HF ratio, R-R, MSSD between R-R intervals, and TP. CCV for HF and LF was also assessed.

Limited studies have assessed HRV in children with disabilities. Time and frequency analysis confirmed the predominance of sympathetic activity in children with disabilities compared to healthy children during sleep (Bouquier, Amand & Van Eecke 2013). Increased sympathetic activity in children with disabilities was associated with a reduction in adaptive abilities of the children’s ANS (Bouquier *et al*. 2013).

To date, a single study in the literature assessed the effects of THR on HRV of children with disabilities (Naidoo *et al*. 2014). The study examined the acute HRV responses to a THR session in children with autism spectrum disorders (ASDs). The findings were increased RMSSD post-THR and reduced LF/HF ratio, which were both suggestive, although not conclusive, of an increase in parasympathetic activity after THR, associated with a calm and relaxed state.

The aim of this article was to determine the effects of THR over a period of six sessions on the HRV in children with various disabilities. The objectives of the study were to examine the effects of THR on the activity of the parasympathetic nervous system over a period of time on children with disabilities, via HRV testing pre- and post-intervention, as well as to determine the effects of THR on the occupational performance of children with disabilities pre- and post-intervention.

## Methods

### Study design

The study used a nonrandomised pre-and post-tests quasi-experimental design to assess the effect of the THR intervention of six sessions on the HRV of children with disabilities. The dependent variable was the HRV measure, whilst the independent variables were the children with disabilities.

### Participants

A convenient sample of 29 children with disabilities (18 boys and 11 girls), with a mean age of 8.69 (±2.22) attending THR sessions, was selected. All participants attended THR sessions at the Ridge Top Equine Centre, KwaZulu-Natal, but were recruited from different schools. The sample was selected because of the easy accessibility to the children in the THR programme by the researchers, as children were all attending at the same riding centre from which permission to conduct the study was granted for. Participants adhered to the following inclusion criteria: children between 5 and 18 years, presenting with a disability as diagnosed by the physician, attending group THR sessions with more than three months experience.

Out of the 29 participants, 12 presented with ASD (41%), 10 with cerebral palsy (34%), 3 with pervasive developmental disorder (10%), 1 with developmental learning disability (3%), 1 with sensory problems (3%), 1 with fanconi syndrome (3%), 1 with blindness (3%) and 1 with Down’s syndrome (3%). Participants were diagnosed by their physicians of the disabilities they presented, however, the severity of the disability was unspecified. All children were verbal and were able to respond (Table 1).

**TABLE 1:** Sample demographics of participants (*n* = 29).

Category	Component	*n*	%
Gender	Male	18	62
	Female	11	38
Race	White	9	31
	Black	8	28
	Indian	5	17
	Coloured	7	24
Mean age (years) (SD)	8.69 (±2.22)	-	-
Mean weight (Kg) (SD)	30.30 (±12.91)	-	-
Mean height (m) (SD)	1.27 (±0.15)	-	-
Disability	Autism spectrum disorder	12	41
	Cerebral palsy	10	34
	Pervasive developmental disorder	3	10
	Developmental learning disability	1	3
	Sensory problems	1	3
	Fanconi syndrome	1	3
	Blindness	1	3
	Down syndrome	1	3

*Source*: Authors’ own work

### Testing procedures and protocol

Consent forms were signed by parents, participants and the owner of the riding school for permission to conduct the study. Parental consent and child assent was obtained on an individual basis. The study was approved (BF074/14) by the Biomedical Research Ethics Committee of the University of KwaZulu-Natal, South Africa.

Participants were familiarised with the placement of the electrodes and Actiheart monitor on their chest during THR sessions two weeks prior to the start of the measurements. Teachers were also shown how to place the electrodes on the participants and were requested to familiarise the children during school hours.

Testing was conducted at the riding centre after the familiarisation sessions and once consent was granted by parents. Testing was performed between 08:30 and 11:00 as that was the scheduled time for riding lessons. Measurements were taken once a week for a period of six weeks using the Actiheart monitor to record data, attaching two electrodes on the chest and the Actiheart. The researcher clarified and answered questions related to the testing procedures.

## Heart rate variability measurement

Actiheart (Cambridge Neurotechnology, Cambridge, UK) monitors were used to measure the inter-beat intervals (IBIs) on participants using the short-term HRV monitoring set-up on the Actiheart software. Two ECG electrodes (Unilect 4040M) were placed on the chest on V2 (4^th^ rib space on the left of the sternum) and V5 (on the 6^th^ rib in line with the anterior axillary line) of the participant.

Measurements were recorded once a week for six weeks and involved three stages in each session. During the first stage, pre-THR measurements were recorded five minutes before riding, for five minutes with participants seated on chairs. The second stage of measurements were recorded during the THR session for 20 to 25 minutes. Lastly, during stage three, post-THR measurements were recorded five minutes after riding, for five minutes with participants seated on chairs.

### Therapeutic horseback riding sessions

Participants were involved in group THR sessions in an outdoor arena conducted by a certified THR instructor. There were two riding groups on each day, and each group consisted of four to six riders per session. Each group was riding once a week, with different groups attending THR each day (Monday to Friday). Each day of the week had one specific THR instructor allocated to conduct the THR session for that day, together with the same side walkers and leaders for each participant. A total of three different THR instructors were available for the week. All three THR instructors followed a similar THR programme each week. Each participant had one volunteer leading the horse and two volunteers as side walkers to assist in THR activities and to ensure proper posture maintenance. The THR instructor stood in the centre of the arena and instructed the horse leaders, riders and side walker of the activities to perform during THR.

The sessions included riding, mounting and dismounting, trotting as well as performing activities such as throwing a ball, extending arms and reaching to touch the horse’s ears or tail during riding.

### Occupational performance questionnaire

The Occupational Performance Questionnaire (OPQ) was utilised to collect pre- and post-THR programme data. The questionnaire was adapted from the, ‘Development of a questionnaire to determine change in the occupational performance of pre-schoolchildren with ASDs receiving Occupational Therapy – Sensory Integration’ (Wallace 2009). The OPQ included information on sleeping patterns, toilet training, impact of the disability on social functions, impact on family members, social interaction, play-time and schooling. The questionnaire was administered to parents/guardians at the first session of THR and post-six weeks of THR. The objective of the questionnaire was to assess if the THR improved the quality of life of the children with disabilities.

## Data analysis

### Heart rate variability

The recorded data for pre-THR, during THR and post-THR session were transferred to the Actiheart software after each session and all data exported to the HRV analysis software at the end of the six sessions for all participants.

R-R intervals (time between QRS complexes), which are the IBIs, were exported as a text file for time domain and spectral HRV analysis using the VarCor PF7 diagnostic device software (DIMEA Group, Olomouc, Czech Republic). The R-R intervals were examined, and all premature ventricular contractions, missing beats, and any artefacts were manually filtered. A set of 300 artefact-free subsequent R-R intervals was obtained. A spectral analysis of HRV was used to assess the ANS activity and was performed using the Fast Fourier Transformation. The spectral analysis incorporated a sliding 256 points Hanning window and a Coarse-Graining Spectral Analysis algorithm (Yamamoto & Hughson 1991). The power spectra was quantified by integrating the area under the power spectral density curve. Two frequency bands were used: low frequency (LF) from 0.05 to 0.15 Hz and HF from 0.15 to 0.50 Hz. The normalised low and HF power (LFnu and HFnu, respectively) were calculated as follows: 100% *×* LF/(LF+HF) and 100% *×* HF/(LF+HF), respectively. Normalisation minimises the effect on the values of LF and HF components of the changes in TP (Taskforce 1996). TP is the variance of all the R-R intervals, with frequency ranges at approximately ≤ 0.4 Hz, measured in ms^2^ (Taskforce 1996).

Repeated measures analysis of variance (ANOVA) was used and applied to each variable for pre-, during and post-THR separately.

### Occupational performance questionnaire

The data were analysed using the Statistical Package for the Social Sciences (Version 21) with significance set at *p* ≤ 0.05. Statistics and tests used were descriptive statistics including means and standard deviations, where applicable, with frequencies represented in tables or graphs. Binomial test was used to test whether the proportion falling in each of the two categories is equal. McNemar test was used to assess for significance of changes; and used to test whether there are differences pre- to post-intervention, with binary measured variables. When the data are categorical (more than two categories), then the Marginal Homogeneity test was used. Chi-square (goodness-of-fit-test) was used on a categorical variable to test whether any of the response options are selected significantly more/less often that the others.

## Results

### Heart rate variability

Both time and frequency domain components of HRV were assessed.

**FIGURE 1:**
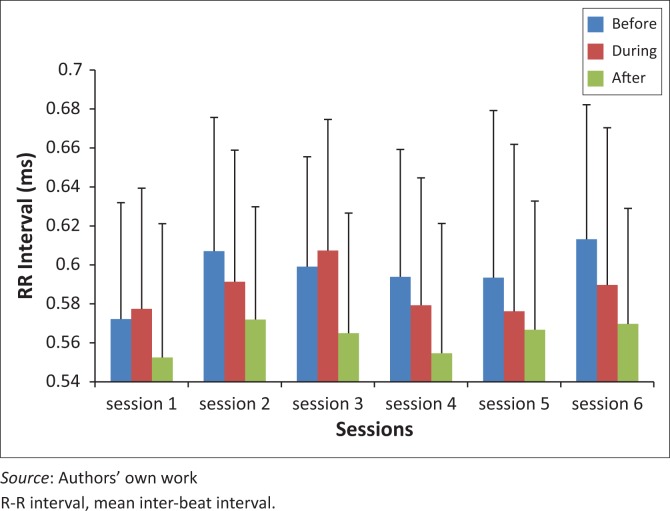
R-R interval before, during and after therapeutic horseback riding (THR) over six sessions.

The average R-R for pre- THR scores were significantly lower at session one than at session two, (*p* = 0.022), session three (*p* = 0.044) and session six (*p* = 0.011). The mean values for the R-R pre-THR were 0.57 (±0.06), 0.61 (±0.69), 0.60 (±0.56) and 0.61 (±0.69) for sessions one, two, three and six, respectively. There were no significant differences for during and post-THR over the six sessions (Figure 1).

**FIGURE 2:**
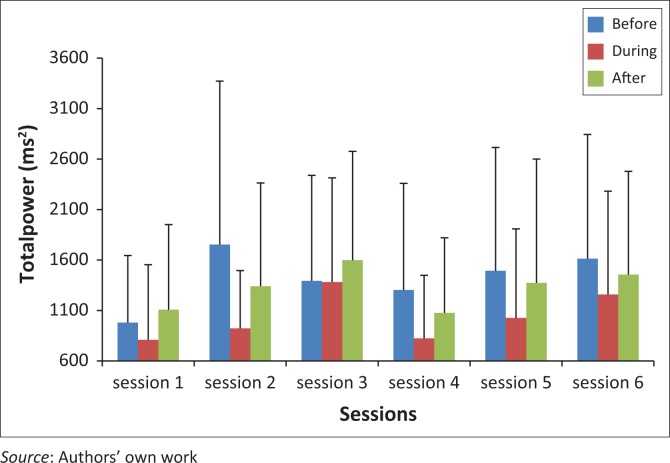
Total power before, during and after therapeutic horseback riding (THR) over six sessions.

TP during THR for session three is significantly higher than sessions one (*p* = 0.044) and two (*p* = 0.024), and session six significantly higher than session four (*p* = 0.045). The mean values for TP during riding were 810.55 (±743.49), 923.19 (±772.13), 1381.88 (±1032.73), 824.01 (±625.26) and 1258.31 (±1024.02) for sessions one, two, three, four and six, respectively. There were no significant differences for pre- and post-THR over the six sessions (Figure 2).

**FIGURE 3:**
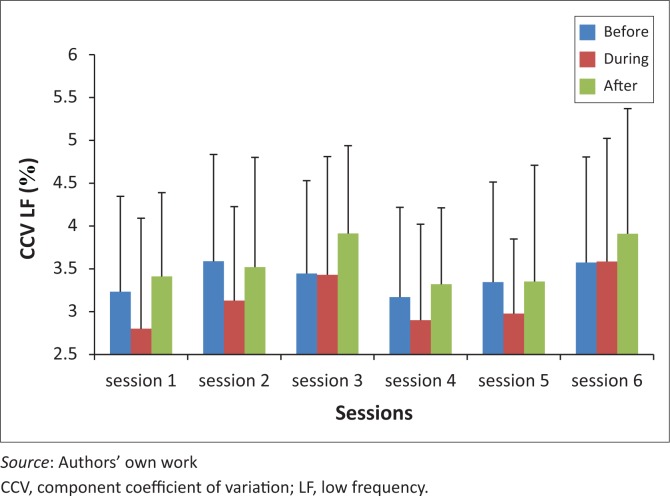
CCV for LF before, during and after therapeutic horseback riding (THR) over six sessions.

CCV for LF during THR is significantly higher in session three than session four (*p* = 0.0006), and session six is significantly higher than sessions one (*p* = 0.022), and four (*p* = 0.011). The mean values for CCV for LF during THR were 2.80 (±1.29), 3.43 (±1.38), 2.90 (±1.12) and 3.59 (±1.44) for sessions one, three, four and six, respectively (Figure 3).

**FIGURE 4:**
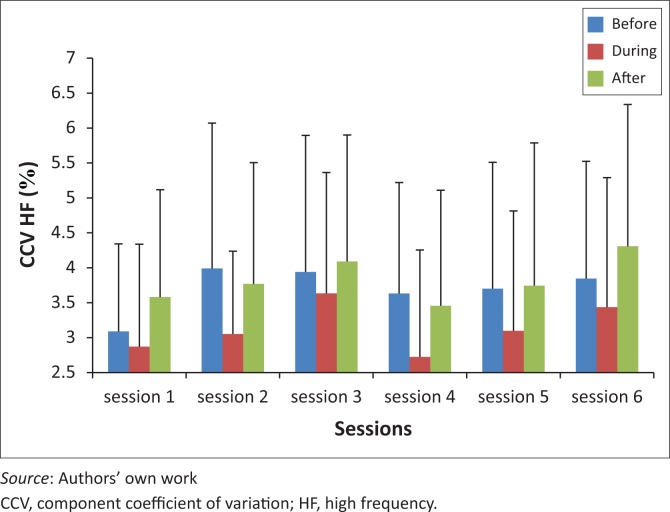
CCV for HF before, during and after therapeutic horseback riding (THR) over six sessions.

There were no significant differences in CCV from session one to session six for pre-, during and post-THR (Figure 4).

**FIGURE 5:**
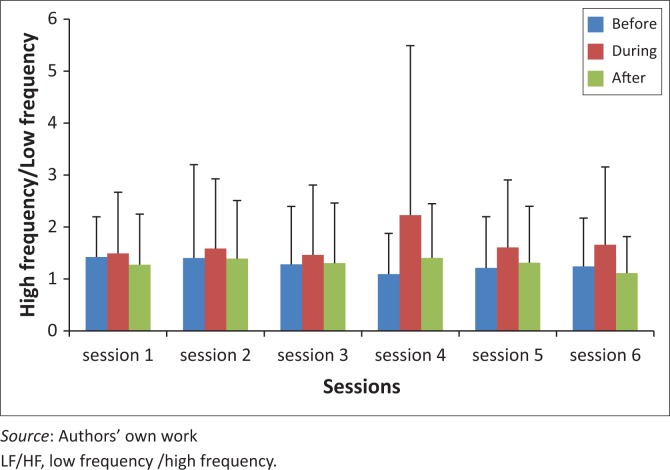
LF/HF before, during and after therapeutic horseback riding (THR) over six sessions.

Figure 5 showed no significant differences in LF/HF from session one to session six for pre-, during and post-THR.

## Occupational performance

The questionnaire evaluated the biological rhythms including toilet training and sleeping pattern, family adjustments, social and play skills, before and after involvement on the THR programme.

### Social functions and family gatherings

A significant proportion of parents reported that they were able to take their child to birthday parties (*p* = 0.008), restaurants (*p* = 0.031), and sustain relationships with other families (*p* = 0.008) before THR. After THR, a significant proportion of parents reported they were able to take their child to birthday parties (*p* = 0.001), restaurants (*p* < 0.005), and sustain relationships with other families (*p* < 0.005). There were no significant reports regarding taking the child to family gatherings before THR, with a significant proportion reporting they are taking the child to the family gatherings after THR (*p* = 0.001).

### Social interaction and play skills

A significant proportion indicated that the child was not over-dependent on his/her parents or clingy after THR (*p* = 0.002). A significant proportion indicated that the child was not able to make friends before THR (*p* = 0.031), but able to make friends after THR (*p* < 0.005). The child was not able to participate in structured group play before THR (*p* = 0.002), but was able to after THR (*p* < 0.005). A significant proportion also indicated that the child was able to play in unfamiliar settings after THR (*p* = 0.002), which was not significant before THR.

## Discussion

The objective of this article was to examine the effects of THR on the activity of the parasympathetic nervous system over a period of time on children with disabilities, via HRV testing pre- and post-intervention. It also sought to determine the effects of THR on the occupational performance of children with disabilities pre- and post-intervention. The primary findings of this study were a significant change in R-R interval pre-THR, CCV LF during THR, and TP during THR, which are discussed in detail in the following section.

### Heart rate variability

With regard to the time domains, the R-R interval reflects the overall HRV, and was lower at session one, particularly for pre-THR, which showed significant increases in the R-R interval scores in sessions two (*p* = 0.022), three (*p* = 0.044) and six (*p* = 0.011) respectively (Figure 1). This may be suggestive of improvements in overall HRV over the six sessions pre-THR. Post-THR, the R-R interval was significantly lower than pre- (*p* < 0.0005) and during (*p* < 0.0005) the THR R-R interval. The decreased R-R interval score post-THR can be associated with increased mental stress (Orsila *et al*. 2008). This could have been as a result of restlessness in children during the measurement because of the nature of the disabilities. Children might have been restless as a result of the excitement and impatience to feed the horses and go back to school, as that is their normal routine. Therefore, as per protocol, to sit down for five minutes could increase the mental stress. There was no change in MSSD, indicating no improvement in parasympathetic activity after the six sessions.

With regard to the frequency domains, there were no significant changes in LF from sessions one to six for pre-, during and post-THR, and no significant differences between pre-, during and post-THR LF scores. This indicates no increase or decrease in sympathetic activity over the six sessions. HF also showed no significant changes over the six sessions and no differences between pre-, during and post-THR scores, indicating no improvement in parasympathetic activity over the six sessions of THR. LF/HF ratio revealed no significant changes over the six sessions of THR (Figure 5). Although not significant, observing the trend of LF/HF over the six sessions of THR, there was a slight reduction in LF/HF over the sessions, predominantly post-THR. Increasing the number of THR sessions could possibly demonstrate a significant reduction in the ratio over the number of sessions, indicating an improvement in parasympathetic activity associated with the relaxation state.

CCV for HF showed no significant change over the six sessions (Figure 4), with the trend slightly increasing with each session. An increase in CCV HF indicates an increase in parasympathetic activity and increase in CCV LF indicates an increase in sympathetic activity (Kurosawa *et al*. 2007). Component coefficient of variance for LF showed a significant increase from session three to session four (*p* = 0.0006), and session six was also significantly higher than session one (*p* = 0.022) and four (*p* = 0.011), during THR (Figure 3). This increased sympathetic activity can be related to the exercises performed during THR. There was no significant change in CCV LF for pre- and post-THR over the six sessions.

There were no significant changes in TP over the six THR sessions for pre- and post-THR. Significance was observed in TP during THR (Figure 2), where session three was significantly greater than sessions one (*p* = 0.044) and two (*p* = 0.024), and session six greater than session four (*p* = 0.045). Increased TP at these points can be associated with increased vagal (parasympathetic) activation, as per study by Taskforce (1996). A marked reduction of TP is associated with sympathetic activation, an increase in TP is associated with vagal activation (Taskforce 1996).

Studies have shown a positive effect of THR on children with disabilities, including improved stereotypic behaviour, hyperactivity (Gabriels *et al*. 2012), motor skills (Gabriels *et al*. 2012; Ionatamishvili *et al*. 2004), improved attention and social motivation (Bass *et al*. 2009). The findings of this article show that there is a change in HRV after a period of six THR sessions for children with disabilities. An increase was noted in R-R interval pre-THR and TP during THR, both suggestive of increased parasympathetic activity, whilst an increase in CCV LF suggested an increased sympathetic activity. Because of the inconsistency between time and frequency domains, no conclusive findings can be reported.

Most studies conducted on THR involved interventions of 10 to 12 weeks to foster significant changes. However, the effect of THR on Down’s syndrome, spina bifida and autism for a seven week THR intervention had shown significant improvements in gross motor function (Winchester *et al*. 2002). The current study was conducted for a period of six sessions with THR performed only once a week by participants. The duration of the sessions might have not been adequate to bring about significant findings on HRV.

### Occupational performance

The OPQ adapted from Wallace (2009) measured different variables to assess if THR intervention has a positive effect on occupational performance of children with disabilities, thus improving quality of life.

There were no problems with biological rhythms including sleeping, toilet training and feeding problems before the THR intervention. There was a significance in family adjustment, with reports indicating that parents were able to take the child to family gatherings after THR (*p* = 0.001). However, parents were still able to take their child to birthday parties and restaurants even before THR intervention. The ability for parents to take the child to family gatherings can be attributed to improved independency. Social interaction reports indicated that the child was not over-dependent or clingy on parents after THR (*p* = 0.002). This could be linked with the interactions the children have during THR sessions, sense of achievement gained through riding and therefore increasing the level of confidence.

These findings are in agreement with the study by Bass *et al.* (2009) which showed improvements in social motivation, less distractibility, less inattention after a 12-week THR intervention in children with ASD. However, another study assessed quality of life of children with cerebral palsy before and after a 10-week THR intervention. The study examined variables such physical well-being, psychological well-being, mood and emotions, parents relations to home life, schooling, and the THR intervention showed no significant improvement in gross motor function, health and quality of life of children with cerebral palsy (Davis *et al*. 2009).

There was an improvement in play skills of children, with the ability to make friends after THR compared to before THR (*p* ≤ 0.005), ability to participate in structured group play (*p* ≤ 0.005) and to play in unfamiliar settings after THR (*p* = 0.002). This could be attributed to activities performed during THR, interaction with other children, riding instructors and with the horses. There was no change in the schooling measures after THR intervention, which is in agreement with the study by Davis *et al*. (2009).

Improvement is evident in selected aspects of occupational performance for children, including social interaction, play skills and family adjustments. However, it should be acknowledged that THR was not the only intervention during the period of data collection, as the questionnaire revealed that 72% of the sample were also undergoing speech therapy, 55.6% physiotherapy, 33.3% occupational therapy and 5.6% applied behavioural analysis. It cannot be concluded that THR alone brought the change in occupational performance, but the improvements could be attributed to a combination of all these methods of treatment including THR.

## Summary

THR of six sessions shows changes in HRV in children with disabilities. However, inconsistency in the results leads to inconclusive findings as to whether sympathetic activity or parasympathetic activity is predominant. For instance, time domain showed an increase in HRV for pre-THR measured by the R-R interval indicating improved vagal activation, whereas frequency domain showed increased sympathetic activity based on CCV LF during THR, and increased parasympathetic activation when assessing TP during THR. However, the quality of life of children was improved in selected aspects.

## Conclusion

Studies have shown a positive effect of THR interventions in children with disabilities including ASDs, cerebral palsy, Down’s syndrome, spina bifida and developmental delay. There is lack of literature pertaining to THR and its effects on the HRV of children with disabilities. This article assessed such effects. The findings showed that a THR intervention of six sessions elicited a change in HRV of children with disabilities. However, the changes obtained were not adequate to make conclusive measures as to whether sympathetic or parasympathetic activity is predominantly increased after the six sessions.

Positively, the THR intervention has been shown to contribute to the improved social interaction, independency, family adjustments and play skills of children with disabilities, hence improving quality of life.

As a result of the lack of literature on the effects of THR on the HRV of children with disabilities, this article provides a basis for further research to be conducted to obtain more information on THR as a tool to improve the parasympathetic activity of children with disabilities.

## Limitations of the study

The sample size for this study was relatively small, therefore results cannot be generalised to a larger population group. Scrupulous science requires that the sample size to be determined according to elicit specific statistical calculations to elecit significant differences. However, because of the limited number of children that were available to attend the THR sessions, the sample size was not calculated but selected based on availability. Further research involving a larger sample is required.

There was also no control group in the study; however the experimental group was acting as their own control comparing pre- and post-THR effects. HRV in children with disabilities should include an experimental group and children with no disabilities as a control group with both groups involved in THR. Finally, HRV in children with disabilities receiving THR (experimental) and those who are not receiving THR (control) would provide more information on HRV changes because of THR. Participants were also involved in other therapeutic interventions, therefore, changes noted in occupational performance after the THR intervention cannot be solely attributed to THR. The changes could be as a result of a combination of all the interventions the participants were involved in, including THR. A study implementing only a THR intervention, without any other form of treatment or therapy would produce more reliable results, although obtaining a statistically significant sample size to participant in a THR programme exclusively, may pose a challenge. However, the HRV changes obtained, which was the primary focus in this study, could be predominantly associated with the THR intervention as the measurements were recorded during the THR sessions.

## References

[CIT0001] Bass M., Duchowny C. & Llabre M, 2009, ‘The effect of therapeutic horseback riding on social functioning in children with autism’, *Journal of Autism Development Disorder* 39, 1261–1267. http://dx.doi.org/10.1007/s10803-009-0734-3 10.1007/s10803-009-0734-3 19350376

[CIT0002] Bilchick K. & Berger R, 2006, ‘Heart rate variability’, *Journal of Cardiovascular and Electrophysiology* 17, 691–694. http://dx.doi.org/10.1111/j.1540-8167.2006.00501.x 10.1111/j.1540-8167.2006.00501.x 16836727

[CIT0003] Bouquier L., Amand M. & Van Eecke D, 2013, ‘Heart rate variability during sleep in children with multiple disabilities’, *Archives de Pediatrie* 20, 1278–1287. http://dx.doi.org/10.1016/j.arcped.2013.09.015 24200422 10.1016/j.arcped.2013.09.015

[CIT0004] Bowes C. & Cook B, 2007, *The value of horse riding and hydrotherapy in the management of severe and complex disability*. In Pope P. (Ed.), Severe and Complex Neurological Disability: Management of the Physical Condition, pp. 197–229. Philadelphia: Elsevier Ltd http://doi:10.1016/B978-0-7506-8825-3.50002-6

[CIT0005] Colhoun H., Underwood S., Fuller J. & Rubens M, 2001, ‘The association of heart rate variability with cardiovascular risk factors and coronay artery calcification’, *Diabetes Care* 24, 1108–1114. http://dx.doi.org/10.2337/diacare.24.6.1108 11375379 10.2337/diacare.24.6.1108

[CIT0006] Davis E., Davies B., Wolfe R., Raadsveld R., Heine B., Thomason P et al., 2009, ‘A randomized controlled trial of the impact of therapeutic horse riding on the quality of life, health and function of children with cerebral palsy’, *Developmental Medicine and Child Neurology* 51, 111–119. http://dx.doi.org/10.1111/j.1469-8749.2008.03245.x 19191844 10.1111/j.1469-8749.2008.03245.x

[CIT0007] Gabriels R., Agnew J., Holt K., Shoffner A., Zhaoxing P., Ruzzano S et al., 2012, ‘Pilot study measuring the effects of therapeutic horseback riding on school-age children and adolescents with autism spectrum disorders’, *Research in Autism Spectrum Disorders* 6, 578–588. http://dx.doi.org/10.1016/j.rasd.2011.09.007

[CIT0008] Homnick T., Henning K., Swain C. & Homnick D, 2015, ‘The effect of therapeutic horseback riding on balance in community-dwelling older adults: A pilot study’, *Journal of Applied Gerontology* 34, 118–126. http://dx.doi.org/10.1177/0733464812467398 25548091 10.1177/0733464812467398

[CIT0009] Ionatamishvili N., Tsverava D., Loriya M., Sheshaberidze E. & Rukhadze M, 2004, ‘Riding therapy as a method of rehabilitation of children with cerebral palsy’, *Human Physiology* 30, 561–656. http://dx.doi.org/10.1023/B:HUMP.0000042613.58352.13 15526448

[CIT0010] Kang K, 2015, ‘Effects of mechanical horseback riding on the balance ability of the elderly’, *Journal of Physical Therapy Science* 27, 2499–2500. http://dx.doi.org/10.1589/jpts.27.2499 26355558 10.1589/jpts.27.2499 PMC4563299

[CIT0011] Kurosawa T., Iwata T., Dakeishi M., Ohno T., Tsukada M. & Marata K, 2007, ‘Interaction between resting pulmonary ventilation function and cardiac function assessed by heart rate variability in young adults’, *Biomedical Research* 28, 205–211. http://dx.doi.org/10.2220/biomedres.28.205 17878600 10.2220/biomedres.28.205

[CIT0012] Lessick M., Shinaver R., Post K., Rivera J. & Lemon B, 2004, ‘Therapeutic horse riding: Exploring this alternative therapy for women with disabilities’, *AWHONN Lifelines* 8, 48–53. 10.1177/1091592304263956 15031888

[CIT0013] Michels N., Sioen I., Clays E., De Buyzere M., Ahiens W., Huybrechts I et al., 2013, ‘Children’s heart rate variability as stress indicators: Association with reported stress and cortisol’, *Biological Pyschology* 94, 433–440. http://dx.doi.org/10.1016/j.biopsycho.2013.08.005 10.1016/j.biopsycho.2013.08.005 24007813

[CIT0014] Miller J. & Alstan A, 2004, ‘Therapeutic riding: An educational tool for children with disabilities as viewed by parents’, *Journal of Southern Agricultural Education Research* 54, 113–123.

[CIT0015] Moodithaya S., & Avadhany S (2009), Comparison of cardiac autonomic activity between pre and post-menopausal women using heart rate variability. *Indian Journal of Physiology and Pharmacology*, 53(3), 277–234. 20329369

[CIT0016] Naidoo R., Nqwena Z., Reimers L., Peters K., Sookan T. & Mckune A, 2014, ‘Acute heart rate variability responses to a therapeutic horseback riding session in children with autsim spectrum disorders: A pilot study’, *Scientific and Educational Journal of Therapeutic Riding* 19, 10–24.

[CIT0017] Orsila R., Virtanen M., Luukkaala T., Tarvainen M., Karjalainen P., Viik J et al., 2008, ‘Perceived mental stress and reactions in heart rate variability – A pilot study among employees of an electronics company’, *International Journal of Occupational Safety and Ergonomics* 14, 275–283. http://dx.doi.org/10.1080/10803548.2008.11076767 18954537 10.1080/10803548.2008.11076767

[CIT0018] Porges S, 1995, ‘Cardiac vagal tone: A physiological index of stress’, *Neuroscience and Biobehavioral reviews* 19, 167–186. http://dx.doi.org/10.1016/0149-7634(94)00066-A 10.1016/0149-7634(94)00066-a 7630578

[CIT0019] Porges S., Doussard-Roosevelt J. & Maiti A, 1994, ‘Vagal tone and the physiological regulation of emotion’, *Society for Research in Child Development* 59, 167–186. http://dx.doi.org/10.2307/1166144 7984159

[CIT0020] Riding for the Disabled Association, 1987, ‘Horse riding for the disabled’, *Australian Journal of Physiotherapy* 33, 202–207. http://dx.doi.org/10.1016/S0004-9514(14)90003-4

[CIT0021] Schroeder E., Whitsel E., Evans G., Prineas R., Chambless L. & Heiss G, 2004, ‘Repeatability of heart rate variability measures’, *Journal of Electrocardiology* 37, 163–172. http://dx.doi.org/10.1016/j.jelectrocard.2004.04.004 15286929 10.1016/j.jelectrocard.2004.04.004

[CIT0022] Snider L., Korner-Bitensky N., Kammann C., Warner S. & Saleh M, 2007, ‘Horseback riding as a therapy for children with cerebral palsy: Is there evidence of its effectiveness?’, *Physical and Occupational Therapy in Paediatrics* 27(2):5–23. 17442652

[CIT0023] Taskforce, 1996, ‘Heart rate variability: Standards of measurements, physiological interpretation and clinical use’, *European Heart Journal* 17, 354–381. 8737210

[CIT0024] Wallace A, 2009, *Development of a questionnaire to determine change in the occupational performance of pre-school children with Autism Spectrum Disorders receiving Occupational Therapy – Sensory Integration*, University of Witwatersrand, Johannesburg.

[CIT0025] Winchester P., Kendall K., Peters H., Sears N. & Winkley T, 2002, ‘The effect of therapeutic horseback riding on gross motor function and gait speed in children who are developmentally delayed’, *Physical and Occupational Therapy in Paediatrics* 22, 37–50. http://dx.doi.org/10.1080/J006v22n03_04 12506820

[CIT0026] Wuang Y., Wang C., Huang M. & Su C, 2010, ‘The effectiveness of simulated horseback riding in children with autism’, *Adapted Physical Activity Quarterly Journal* 27, 113–126. 10.1123/apaq.27.2.113 20440023

[CIT0027] Xie Y., Jiao Q., Guo S., Wang F., Cao J. & Zang Z, 2005, ‘Role of parasympathetic overactivity in water immersion stress-induced gastric mucosal lesion in rat’, *Journal of Applied Physiology* 99, 2416–2422. http://dx.doi.org/10.1152/japplphysiol.00267.2005 16051715 10.1152/japplphysiol.00267.2005

[CIT0028] Yamamoto Y. & Hughson R, 1991, ‘Coarse-graining spectral analysis: New method for studying heart rate variability’, *Journal of Applied Physiology* 7, 1143–1150. 10.1152/jappl.1991.71.3.1143 1757311

